# Raman Diffusion-Ordered Spectroscopy

**DOI:** 10.1021/acs.jpca.3c03232

**Published:** 2023-09-01

**Authors:** Robert W. Schmidt, Giulia Giubertoni, Federico Caporaletti, Paul Kolpakov, Noushine Shahidzadeh, Freek Ariese, Sander Woutersen

**Affiliations:** † Vrije Universiteit Amsterdam, De Boelelaan 1105, 1081HV Amsterdam, The Netherlands; ‡ University of Amsterdam, Science Park 904, 1098XH Amsterdam, The Netherlands; § Université Libre de Bruxelles, Av. Franklin Roosevelt 50, 1050 Bruxelles, Belgium

## Abstract

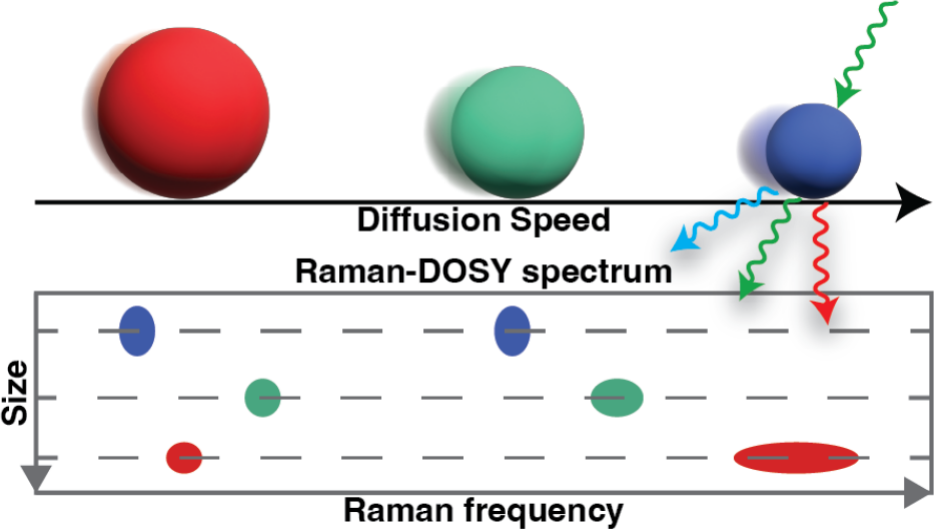

The Stokes–Einstein relation, which relates the diffusion coefficient of a molecule to its hydrodynamic radius, is commonly used to determine molecular sizes in chemical analysis methods. Here, we combine the size sensitivity of such diffusion-based methods with the structure sensitivity of Raman spectroscopy by performing Raman diffusion-ordered spectroscopy (Raman-DOSY). The core of the Raman-DOSY setup is a flow cell with a Y-shaped channel containing two inlets: one for the sample solution and one for the pure solvent. The two liquids are injected at the same flow rate, giving rise to two parallel laminar flows in the channel. After the flow stops, the solute molecules diffuse from the solution-filled half of the channel into the solvent-filled half at a rate determined by their hydrodynamic radius. The arrival of the solute molecules in the solvent-filled half of the channel is recorded in a spectrally resolved manner by Raman microspectroscopy. From the time series of Raman spectra, a two-dimensional Raman-DOSY spectrum is obtained, which has the Raman frequency on one axis and the diffusion coefficient (or equivalently, hydrodynamic radius) on the other. In this way, Raman-DOSY spectrally resolves overlapping Raman peaks arising from molecules of different sizes. We demonstrate Raman-DOSY on samples containing up to three compounds and derive the diffusion coefficients of small molecules, proteins, and supramolecules (micelles), illustrating the versatility of Raman-DOSY. Raman-DOSY is label-free and does not require deuterated solvents and can thus be applied to samples and matrices that might be difficult to investigate with other diffusion-based spectroscopy methods.

## Introduction

The diffusion of molecules is a fundamental mode of mass transport,^1^ and the Stokes–Einstein equation allows us to correlate the diffusion coefficient of a molecule or particle with its hydrodynamic radius.^2,3^ In practice, this relation is often used to estimate molecular size or to observe changes in molecular size due to aggregation^4−8^ or ligand binding.^9,10^ Such processes are often difficult to observe with spectroscopic methods because the aggregation or ligand binding may cause only a small change in the spectrum, which in addition might not be correlated in a straightforward manner to the size of the supramolecules.^11^

Nuclear magnetic resonance diffusion-ordered spectroscopy (NMR-DOSY) successfully combines the size sensitivity of the diffusion coefficient with the structural sensitivity of molecular spectroscopy and is a widely used technique to simultaneously determine the size and chemical structure of molecules.^12−24^ Here, we present the Raman analogue of this method. Raman spectroscopy is an excellent tool for analyzing the bond vibrations within a molecule, making it possible to identify a wide range of compounds with a high degree of certainty.^25−29^ The label-free nature of the method allows us to study the compounds in their native form and dissolved in aqueous solutions. However, the Raman spectrum generally provides little information about the size of molecules or aggregates. To address this issue, we took the experience that we gained from our previous work on infrared-based diffusion ordered spectroscopy (IR-DOSY)^30,31^ and designed a Raman-based DOSY method. Similar to an NMR-DOSY spectrum, a Raman-DOSY spectrum is a two-dimensional spectrum with Raman frequency on one axis and diffusion coefficient on the other and thus provides simultaneous information on the chemical structure and the size of a compound or of a mixture of compounds. Besides forming the spectroscopic complement to IR-DOSY, Raman-DOSY has the practical advantage that there is no need to use deuterated solvents and that the sample cell can be made from glass windows instead of infrared-transparent materials that are generally more expensive. In the case of mixed samples, Raman-DOSY makes it possible to characterize the chemical structure of the molecules in the mixture by separating the Raman peaks into subsets, each of which is associated with a different diffusion coefficient and hence, size.

## Methods

### Experiment

*Sample Preparation*. The mixtures were made by dissolving 8 wt % sodium dodecyl sulfate (SDS, Sigma-Aldrich), 0.5 wt % cytochrome *c* of bovine heart (Fluka), and 3% (v/v) acetonitrile (Sigma-Aldrich) in Milli-Q water.

**Figure 1:**
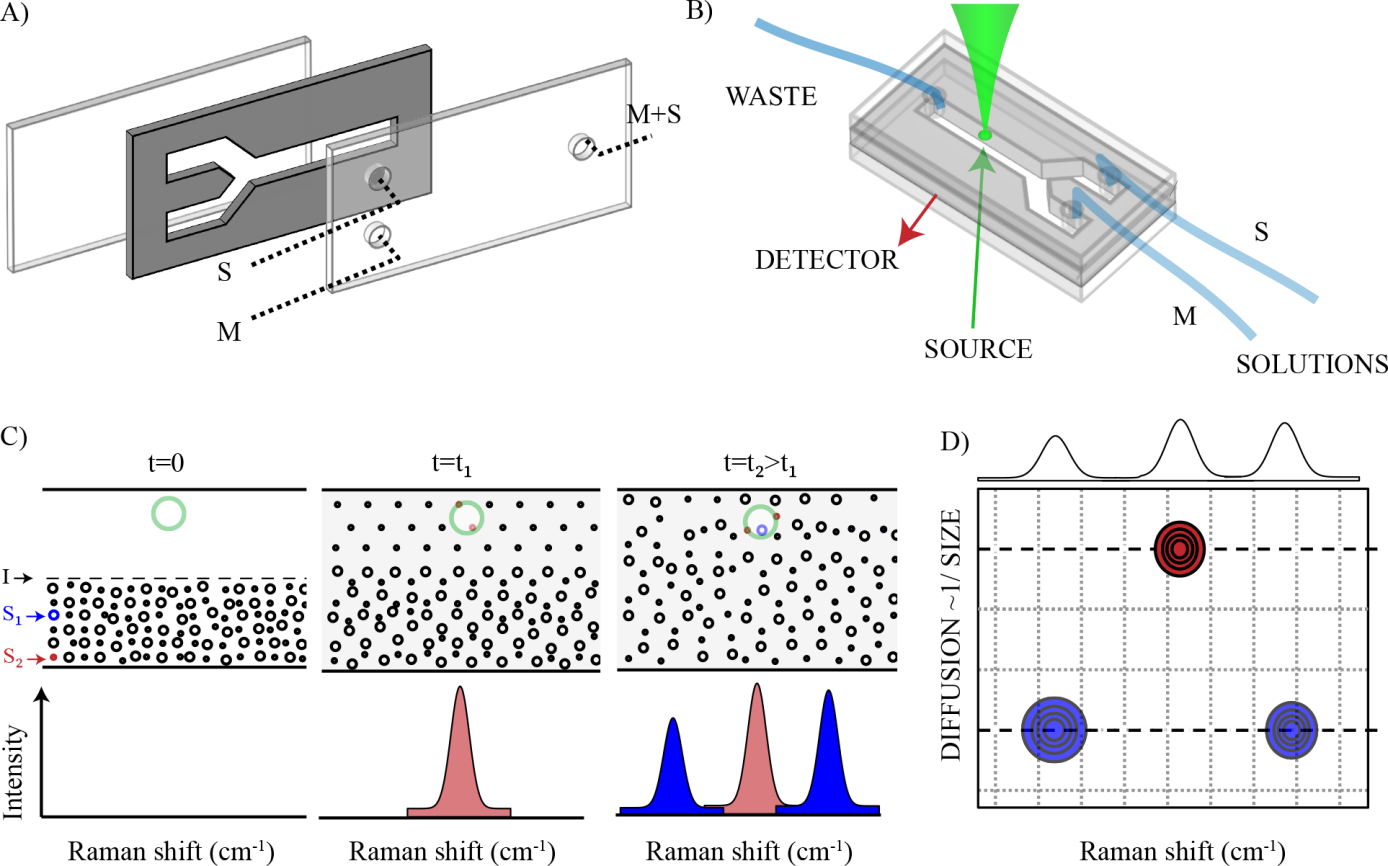
Raman diffusion-ordered spectroscopy. (A) Components of the Raman-DOSY sample cell. (B) Experimental implementation of Raman-DOSY. (C) Operation principle: the sample mixture (M) and pure solvent (S) are pumped into a channel (4 mm wide). In this example, the sample solution contains two molecular species, *S*_1_ and *S*_2_, of different sizes. The flow rates of the sample solution and solvent are the same; therefore, the interface, *I*, between the two liquids is a line at the midpoint of the channel. When measuring the Raman intensity at the far edge of the solvent-filled half (Raman laser focus indicated by green circle), a time-dependent spectrum is observed, in which the Raman peaks of *S*_2_ appear before those of *S*_1_ due to the larger diffusion coefficient of the smaller species. (D) Schematic Raman-DOSY plot obtained from the time-dependent data, in which the Raman spectra of the different species are ordered according to their diffusion coefficient.

*Raman-DOSY Setup*. In all experiments, the solvent and mixture were injected using 1 mL syringes and a syringe pump (Harvard, model 22 MA1 55-2226) at a flow rate of 25 μL/min to avoid turbulent flow. To perform Raman spectroscopy, a Raman-DOSY cell with a 4 mm wide channel was used (Figure 1B). Krytox silicone paste (Chemours) was used to waterproof the glass–Teflon interface. *Raman Microscopy*. Confocal Raman microscopy experiments were performed on a WITec inverted microscope system (Alpha 300 RI), equipped with a 10× Nikon objective (TU Plan Fluor EPI, WD 17.5 mm, NA 0.3). The samples were excited with a 532 nm laser (WITec) with 10 mW power at the sample for both experiments. The backscattered light was analyzed with a UHTS spectrometer from WITec with a 600 grooves/mm diffraction grating and measured with a Newton camera (Andor). The WITec algorithm was used to remove cosmic rays and the fluorescence baseline after acquisition. The time series was continuously acquired with an exposure time of 10 s for the samples with dual and triple components.

### Data Analysis

*Spectral Processing*. The Raman spectra were further preprocessed in MATLAB R2021b (The MathWorks Inc., Natick) by truncating the spectra to only include the region of interest of 2200–3000 cm^–1^ and 1500–3000 cm^–1^ for the dual and triple mixtures, respectively. The first spectrum of the time series was used as a blank spectrum and subtracted from the whole data set, including the first spectrum, to remove the Raman peaks of water and glass. The blank spectrum was smoothed by a moving average filter with a window size of 5 before subtraction. Each spectrum was additionally smoothed by a Savitzky–Golay filter with a quadratic polynomial function and a window size of 10 to reduce the noise. *Time-Dependent Concentration at the Edge of the Channel*. To analyze the data, we need an explicit expression for the time-dependent concentration of each of the compounds at the far edge of the initially solvent-filled half of the channel. This expression can be obtained by solving the diffusion equation. Sufficiently far from the entrance and exit holes, the diffusion is effectively one-dimensional, with the initial (*t* = 0) concentration profile given by a step function (Figure 1C). Introducing a dimensionless time variable τ = *Dt*/*L*^2^ (with *D* the diffusion coefficient and *L* the channel width), the time-dependent concentration at *x* = *L*/2 (viz. at the edge of the channel), this is given by *c*(*t*) = *C*(*Dt*/*L*^2^) = *C*(τ) with *C*(τ) a function that does not depend on *D* or *L*. Full summation expressions for *C* can be found in ref (30), but for practical purposes, we can achieve a relative precision of 10^–8^ (with respect to the exact solution) at all times using the expression
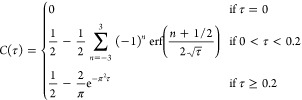
1where erf(*x*) is the Gaussian error function.

## Results

### Design and Operating Principle

The experiments are performed with a flow cell constructed out of a glass window at the bottom, a CaF_2_ window at the top, and a Teflon spacer (thickness 250 μm) in the middle that forms the flow channel. The upper window has three holes that are connected to tubing to inject sample mixture (M) and pure solvent (S) into the flow cell and another to remove the mixed solutions (M + S) from the flow cell into the waste container (Figure 1A,B). The spacer has a Y-shaped cutout to ensure that the two solutions create a liquid–liquid interface line (laminar flow, Reynolds number ≈0.005) in the center of the flow cell. Figure 1C shows the principle of the experiment for a mixed solution containing two compounds of different sizes. The flow is created by injecting the mixture and solvent at a constant flow rate using a double syringe pump. The two liquids create an interface (I) in the middle of the flow channel, and we position the laser focus (green circle in Figure 1C) at the edge of the solvent-filled half of the channel. Then the flow is stopped, and a time series of Raman spectra is measured. At the beginning of the measurement (*t* = 0) the Raman spectrum (measured at the edge of the channel) contains only solvent peaks. With increasing waiting time, the solute molecules diffuse into the solvent-filled half of the cell. The smallest compound (S_2_, red in Figure 1C) of the mixture diffuses most rapidly into the solvent-filled half of the channel and first appears in the spectrum. This is followed by the larger compound (S_1_, blue in Figure 1C) which diffuses at a slower rate into the laser probing area. The spectrum measured at each time point is a combination of faster and slower compounds, and from a global analysis of the two-dimensional data set, we obtain a two-dimensional DOSY plot, with Raman frequency on one axis and diffusion coefficient on the other (Figure 1D).

### Two-Component Raman-DOSY

**Figure 2:**
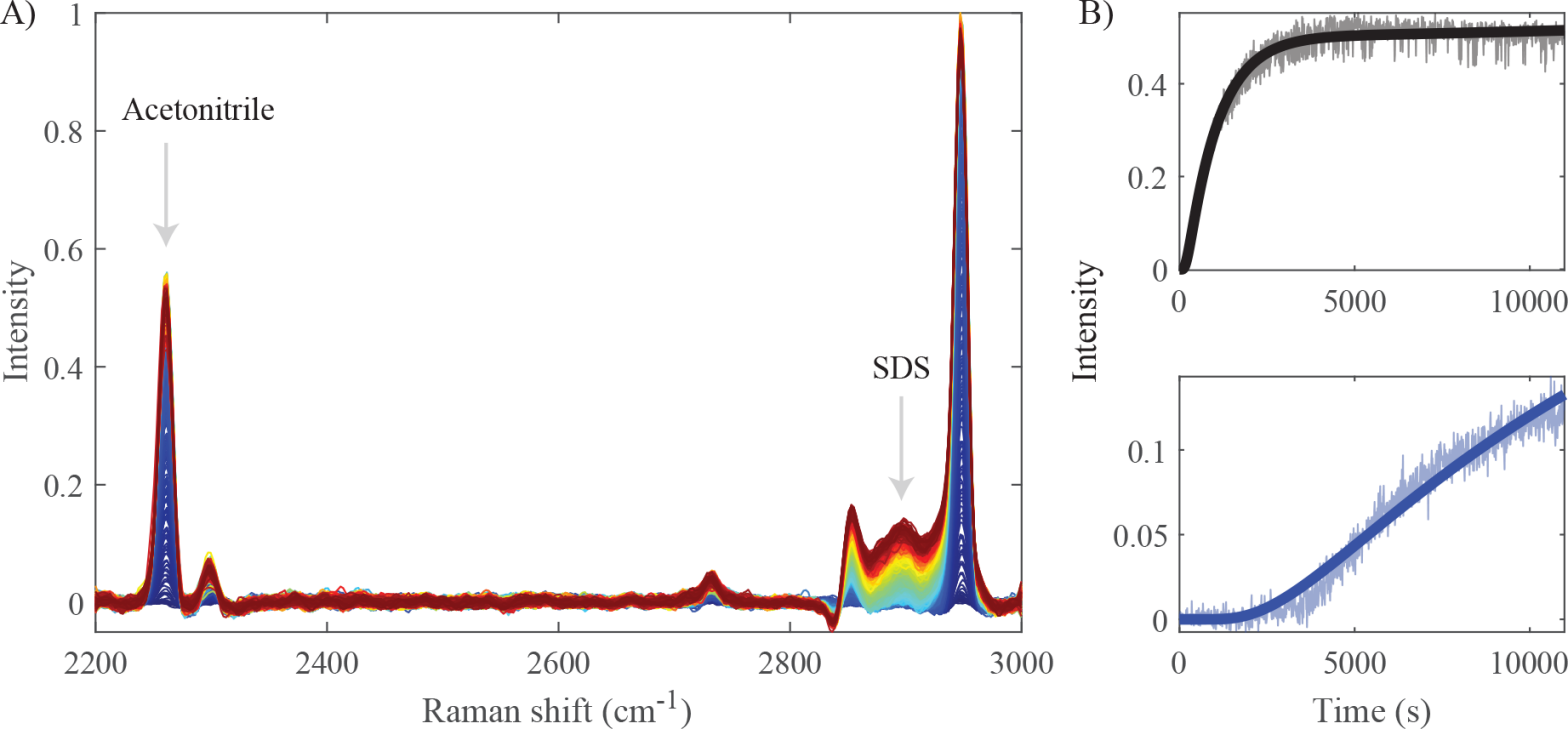
Raman diffusion-ordered spectroscopy of a two-component mixed solution. (A) Time series of Raman spectra (color-coded from blue to red) of a mixed solution of acetonitrile and SDS in water. (B) Raman intensity at 2260 and 2900 cm^–1^ as a function of time after stopping the flow. The solid curves are least-squares fits of the solution to the diffusion equation (eq 1) for acetonitrile (*D* = (1.8 ± 0.6) × 10^–5^ cm^2^/s, black curve) and SDS micelles (*D* = (1.3 ± 0.5) × 10^–6^ cm^2^/s, blue curve).

We first demonstrate the setup with a mixed aqueous solution of acetonitrile and sodium dodecyl sulfate (SDS) micelles. After injecting the solution and solvent and stopping the flow, we record a full Raman spectrum every 10 s and crop it to the frequency range 2200–3000 cm^–1^ that contains major Raman peaks of the two compounds. Figure 2A shows the time-dependent Raman spectrum, and Figure 2B shows the Raman intensities at the frequencies of the acetonitrile CN-stretching mode and the SDS CH-stretching mode (2260 and 2900 cm^–1^, respectively) as a function of time, highlighting the different time dependencies due to the difference in diffusion coefficient of acetonitrile and SDS. The diffusion coefficients are determined by least-squares fitting the time-dependent data to the solution of the diffusion equation (eq 1; the fits are shown as solid curves). Acetonitrile, the smaller compound in the mixture, has a diffusion coefficient *D* of (1.8 ± 0.6) × 10^–5^ cm^2^/s, while for SDS we obtain *D* = (1.3 ± 0.5) × 10^–6^ cm^2^/s. For the SDS micelles, the fit curve does not reproduce the experimental data perfectly. We attribute this discrepancy to the fact that SDS can be present both as monomers and as micelles. In the sample solution, the SDS concentration is well above the critical micelle concentration (CMC) so most SDS is present as micelles, but the first SDS micelles to diffuse into the initially solvent-filled half of the channel will experience a local SDS concentration below the CMC and therefore partly dissociate into monomers, whereas at a later stage (when the local SDS concentration has become larger than the CMC) this will no longer happen. As a consequence, the initial lag phase is somewhat longer than predicted by the diffusion model (which assumes all SDS to be present as micelles), and the plateau is reached somewhat earlier than predicted by the diffusion model.

Using these two diffusion coefficients, we globally fit the full time-dependent Raman data set at all frequencies to create a Raman-DOSY spectrum. The time- and frequency-dependent Raman intensity *S* measured at the far edge of the channel is fitted to the function^30^
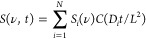
2where *N* is the number of species in the sample (in this case *N* = 2), *S*_*i*_(ν) is the Raman spectrum of species number *i*, *D*_*i*_ is the diffusion coefficient of species *i*, and *C*(τ) is a function (given in eq 1) depending only on the reduced time τ = *Dt*/*L*^2^. From the fit, we obtain the spectra *S*_1,2_(ν) of the two compounds in the mixed solution. To obtain a DOSY spectrum from the fit results, we use a procedure similar to that in ref (18): the 2D-DOSY spectrum *I*(ν,*D*) is obtained by multiplying the spectral amplitude *S*_*i*_(ν) with the appropriate probability distribution for *D*_*i*_:
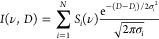
3where *N* is the number of species and σ_*i*_ are the uncertainties in the diffusion coefficients *D*_*i*_ obtained from the least-squares fits.

**Figure 3:**
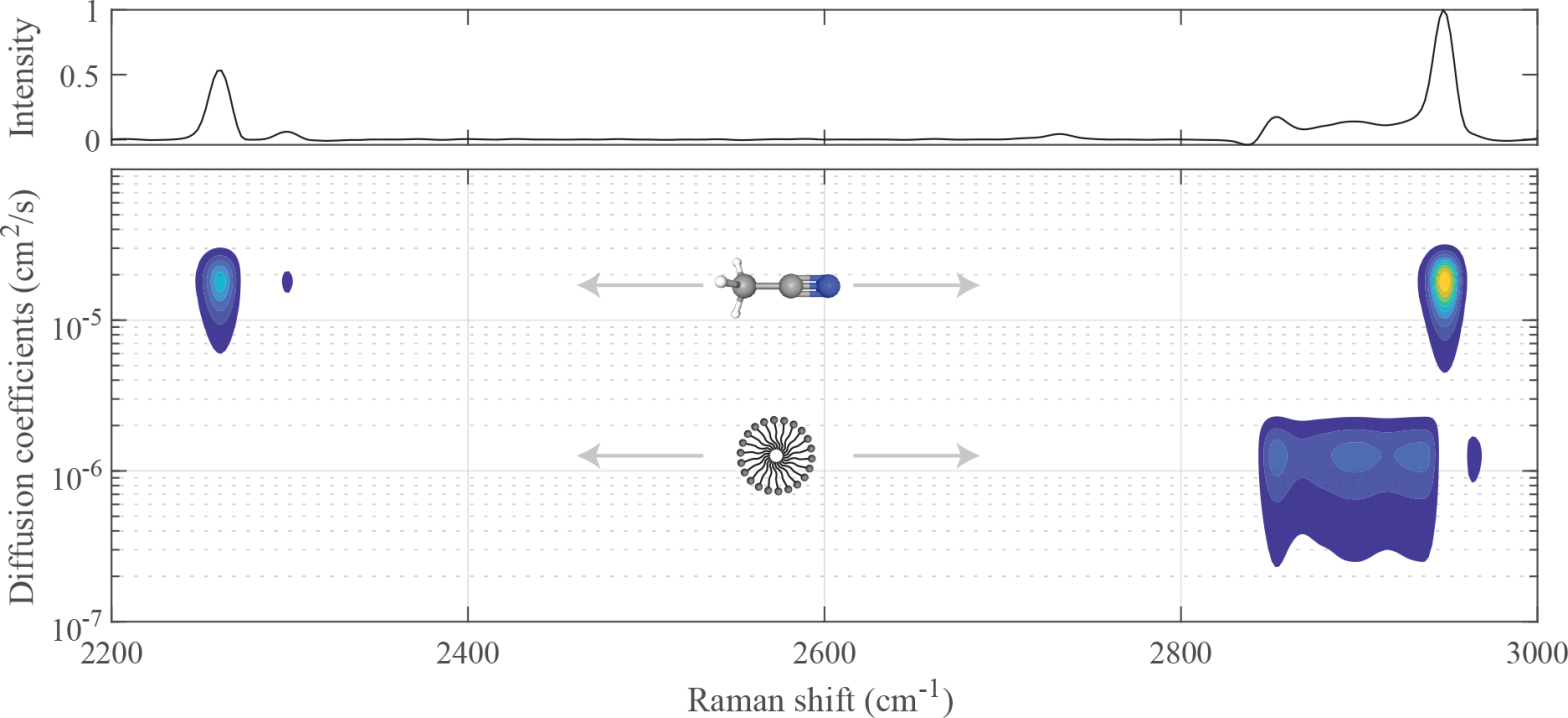
Conventional Raman spectrum (top) and Raman-DOSY spectrum (bottom panel) of the mixed solution of acetonitrile and SDS. The structures of the compounds are shown with the corresponding diffusion coefficients.

The resulting Raman-DOSY spectrum is shown in Figure 3. In this spectrum, the spectral bands are separated into two rows, vertically positioned at the diffusion coefficients of the two components. The upper row has three peaks, at 2261, 2300, and at 2948 cm^–1^, which are due to acetonitrile.^32,33^ The bottom row has a broad peak between 2840 and 2947 cm^–1^ and a small peak at 2974 cm^–1^, both of which are consistent with SDS.^34,35^ In the conventional Raman spectrum of the mixture (top panel of Figure 3), the latter peak overlaps with the peak of acetonitrile at 2948 cm^–1^, but in the Raman-DOSY spectrum, these two peaks are cleanly separated. For comparison, the Raman spectra of the individual compounds are shown in Figure S1.

From the diffusion coefficients we can estimate the molecular sizes.^36,37^ Using the Stokes–Einstein equation (with η = 0.001 Pa·s and *T* = 293.15 K), we obtain an estimated hydrodynamic radius of 0.089–0.178 nm for acetonitrile and 1.34–2.15 nm for SDS. The radius of acetonitrile in water matches the predicted literature value of 0.143 nm (*D* = 1.53 × 10^–9^ m^2^/s, η = 0.001 Pa·s, and *T* = 298 K for the MCM boosting method estimation).^38^ At the concentration used in our experiment, SDS self-assembles into micelles (the critical micelle concentration is 8.5 mmol/L),^39^ and the hydrodynamic micelle radius of ∼1.65 nm obtained from our experiment agrees well with the previously established value of 1.75 nm.^40^

### Three-Component Raman-DOSY

**Figure 4:**
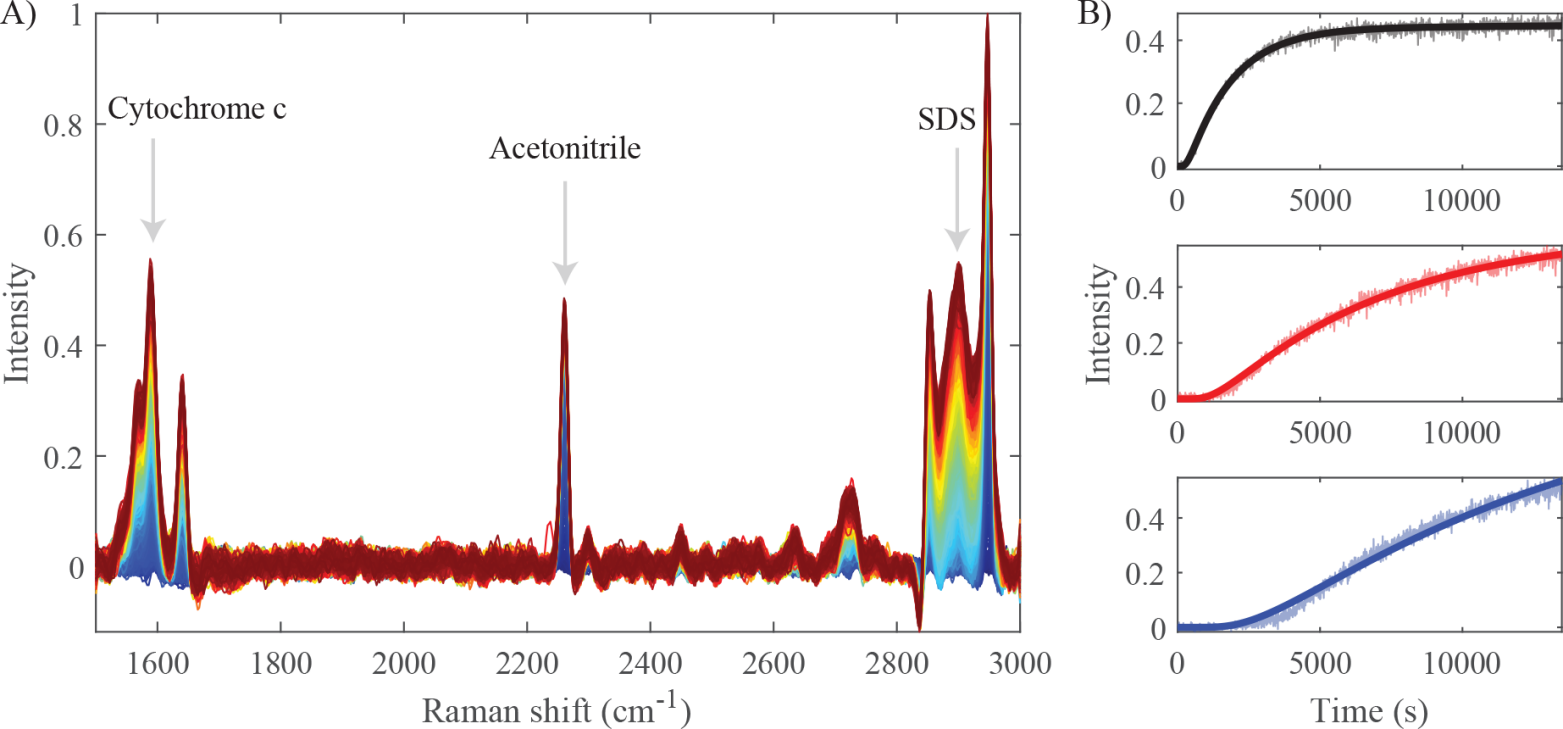
Raman diffusion-ordered spectroscopy of a mixed solution of cytochrome *c*, acetonitrile, and SDS. (A) Time series of Raman spectra (color-coded from blue to red). The arrows in the graph indicate the Raman frequencies of the compounds used to determine their diffusion coefficients (1590, 2260, and 2900 cm^–1^). (B) Time-dependent Raman intensities at these three frequencies and least-squares fits to eq 1 (solid curves). The diffusion coefficients obtained from the fits are (1.0 ± 0.2) × 10^–5^ cm^2^/s for acetonitrile (black), (2.7 ± 0.5) × 10^–6^ cm^2^/s for cytochrome *c* (red), and (1.3 ± 0.3) × 10^–6^ cm^2^/s for SDS micelles (blue).

**Figure 5:**
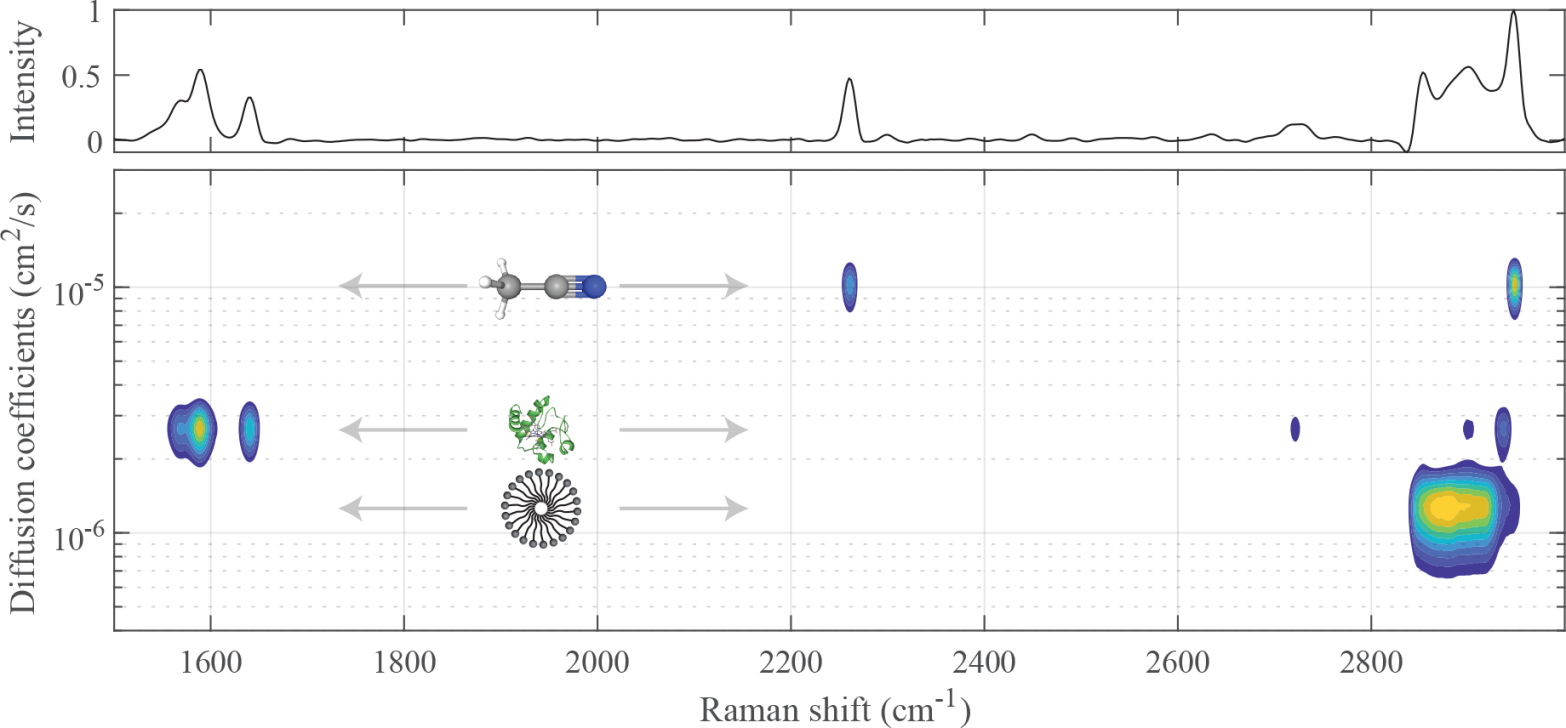
Conventional Raman spectrum (top) and Raman-DOSY spectrum (bottom) of a mixed solution of acetonitrile, cytochrome *c*, and SDS micelles, including the structures of the compounds at their respective diffusion coefficient values.

In the second experiment, the mixture was expanded with a third component, cytochrome *c*, a small, commonly occurring heme protein.^41^ The analyzed wavenumber range was also expanded, starting at 1500 cm^–1^ to also include the cytochrome *c* specific peak at 1590 cm^–1^. Figure 4A shows the Raman spectra as a time series, and Figure 4B shows the relative Raman intensities of the three compounds as a function of time. Cytochrome *c* is larger than acetonitrile but smaller than the SDS micelles, as can be seen directly from the lag phase and rise time in the graph. By least-squares fitting eq 1 to the time-dependent data, we obtain diffusion coefficients (1.0 ± 0.2) × 10^–5^ cm^2^/s for acetonitrile, (2.7 ± 0.5) × 10^–6^ cm^2^/s for cytochrome *c*, and (1.3 ± 0.3) × 10^–6^ cm^2^/s for the SDS micelles (see the previous section for a discussion regarding the imperfect fit to the SDS data). In the Raman-DOSY spectrum (Figure 5), the compounds are separated into three rows. In the upper row, the spectral bands are centered at 2260 and 2948 cm^–1^, which correspond to Raman shifts of acetonitrile. In the middle row, we observe a shoulder peak at 1588 cm^–1^ and single peaks at 1640, 2722, 2853, 2900, 2937, and 2952 cm^–1^, which correspond to cytochrome *c*.^42,43^ The bottom row contains a broad peak at 2875 cm^–1^ and a small peak at 2974 cm^–1^, which are consistent with SDS (see the reference spectra in Figure S1). All three compounds have peaks in the region between 2800 and 3000 cm^–1^, which is generally highly congested due to the overlap of the alkyl CH stretching modes, but in the Raman-DOSY plot, they are neatly resolved (Figure 5). Acetonitrile diffuses more slowly in the triple mixture (1.8 × 10^–5^ cm^2^/s) than in the double mixture (1.0 × 10^–5^ cm^2^/s), possibly due to crowding by the cytochrome *c*, making the movement of acetonitrile more difficult. The contours of the SDS micelles in Figure 5 are slightly different from the ones in the two-component mixture, a difference that we tentatively attribute to cross talk between the compounds in the global fit, as the cytochrome *c* Raman peak is rather close to that of SDS.

## Discussion

The above results show that Raman-DOSY can be used to characterize the chemical structure (from the Raman frequencies) and size (from the diffusion coefficient) of a compound or a mixture of compounds in solution. In these experiments, the number of species in the solutions was known beforehand, and we could obtain their diffusion coefficients by analyzing the time-dependent Raman intensity at compound-specific frequencies. In general, (i) the number of compounds might not be known, and (ii) there might not be isolated Raman peaks for all of the compounds. The first problem can be solved by performing singular-value decomposition (SVD) of the raw data. In fact, SVDs of the raw data of Figures 2 and 4 directly show the number of compounds in these solutions (see Figures S2 and S3). The second problem can be solved by performing a global least-squares fit, in which the diffusion constants in eq 2 are treated as free parameters (the number *N* of species having been obtained from the SVD analysis). With an increasing number of compounds in a mixture this approach will, sooner or later, become difficult. In NMR-DOSY, this problem has been solved by applying advanced multivariate algorithms (such as MCR,^44^ SCORE,^45^ DECRA,^46^ and PALMA^47^) that can deconvolute the overlapping spectra very efficiently, and we believe that with minor modifications these algorithms may also be used for Raman-DOSY.

The time required for a Raman DOSY measurement is determined by the diffusion coefficient *D* of the molecules under study and the width *L* of the channel, and is given roughly by *L*^2^/*D*. In the measurements shown above the measurement time was about 3 h. Longer measurement times than we have used are possible, although at some point laser stability and laser-induced degradation might become a limitation, especially for larger molecules that may take more time to diffuse through the solvent channel into the probing area of the laser. This problem can be solved by reducing the channel width (the characteristic diffusion time is *L*^2^/*D*, so reducing the channel width by a factor 10 reduces the required measurement time by a factor 100) or by positioning the laser focus closer to the liquid–liquid interface. The measurement time of large molecules can be further decreased by applying electrophoresis to the flow cell, which separates larger molecules (proteins and DNA) according to their size and charge.

In the experiments reported here, the integration time was 10 s, but for compounds with small Raman cross sections, longer integration times may be required. With respect to the integration time and sensitivity, the same considerations apply as for conventional Raman spectroscopy: the optimal integration time and laser power depend on the Raman cross section of the molecules under study. The experimental parameters can easily be optimized because one can check beforehand if a Raman DOSY measurement will be successful in terms of signal-to-noise by simply measuring a Raman spectrum in the filled half of the channel (taking into account that the signal amplitude in the Raman DOSY spectrum is reduced by a factor 2 compared to the Raman spectrum of the sample solution due to the dilution in the channel).

When compounds diffuse into the focal volume of the laser, a baseline drift of the Raman spectra may occur. This is a common phenomenon for highly fluorescent biological molecules, and many baseline removal methods have been developed.^48−50^ However, a robust fluorescence baseline removal method is a necessity for the time series data set, where the peak intensity is used for the fitting. Therefore, an optimal baseline correction that does not influence peak intensities is essential. Besides optimizing the baseline removal parameters, one can apply a smoothing filter along the time dimension to even out single baseline changes or one could change the laser excitation wavelength to the NIR range. The latter will reduce the fluorescence interference because fewer compounds will be excited at that wavelength.^51^ On the other hand, selecting an excitation laser wavelength close to the absorption of the compound may also have its advantages because it can give rise to a resonance Raman effect. The large gain in the Raman signal increases the sensitivity toward molecules in the channel, making it possible to analyze compounds at low concentrations. In order to avoid fluorescence, excitation close to a higher electronic transition or in the deep-UV range would be preferred.^52^ Finally, the spectral acquisition time (and hence the temporal resolution in the case of rapidly diffusing compounds) can be significantly improved by using multiplex-stimulated Raman scattering (which is also insensitive to fluorescence), with acquisition times that can be less than a few milliseconds.^53^

NMR peaks are generally narrower and less likely to overlap than peaks in a Raman spectrum, so the spectral resolving power of NMR DOSY is certainly better than that of Raman DOSY. However, Raman DOSY has its advantages: no deuterated solvents are required, measuring paramagnetic compounds is no problem, and Raman spectrometers are less costly than NMR spectrometers. Raman DOSY has a spectral resolving power similar to that of IR DOSY, on which we reported previously,^30^ and the two methods are somewhat complementary because normal modes that have low IR cross sections may still have significant Raman cross sections, and vice versa.^54^ Like NMR, IR-DOSY often requires deuterated solvents, while Raman DOSY does not, and in Raman-DOSY we can achieve somewhat better spatial resolution than IR DOSY (in which a slit is used to spatially select the edge of the solvent-filled part of the channel in the flow cell).

## Conclusions and Outlook

Raman scattering microscopy is a nondestructive and label-free method for analyzing the chemical structures of molecules, and when combined with diffusion-ordered spectroscopy, it becomes a simple and cost-effective tool that can additionally characterize the size of molecules or aggregates. Raman spectroscopy characterizes the functional groups of molecules and the backbone vibrations of polymers. By selecting an excitation laser wavelength close to the absorption wavelength of the molecule, one can additionally induce a resonance Raman effect, which increases the Raman signal and allows for the analysis of molecules with lower concentrations in the flow cell.

The separation power of Raman-DOSY is similar to that of NMR-DOSY, but Raman spectroscopy provides significantly less structural information than NMR. The separation power might be increased by adding electrophoresis to the flow cell, making it possible to analyze larger molecules in a reasonable time. We demonstrated the potential of Raman-DOSY to measure the diffusion coefficients of individual compounds in mixtures with two or three compounds. Mixtures with more compounds can be measured by applying more advanced analysis methods, notably the ones that have been developed for NMR-DOSY, and similarly to multidimensional NMR-DOSY, it should also be possible to measure multidimensional-Raman DOSY by combining multidimensional Raman spectroscopy^55−57^ with a DOSY flow cell.

We believe that Raman-DOSY may find applications in the biomedical field to analyze the sizes of proteins in physiological solutions. Another application could be in the polymer research field^28,29^ because polymers have narrow and intense Raman peaks in the fingerprint region and intense CH-stretch vibrations of the polymer backbone in the Raman spectra. We think that Raman-DOSY can be a useful complement to NMR-DOSY: it is comparatively cost-effective, does not require deuterated solvents, and opens up the possibility of analyzing samples that are difficult to measure with NMR, such as paramagnetic compounds or compounds that have strongly overlapping NMR spectra.

## Supporting Information Available

Raman spectra of acetonitrile, SDS, and cytochrome *c* (PDF)
