# Supplementary material for: Raman Diffusion-Ordered Spectroscopy
Source: J Phys Chem A. 2023 Sep 1;127(36):7638–45. doi: 10.1021/acs.jpca.3c03232 (PMC10510375; doi:10.1021/acs.jpca.3c03232)
Supplement: Supplementary file 1 — jp3c03232_si_001.pdf [file jp3c03232_si_001.pdf]

Raman Diffusion-Ordered Spectroscopy: Supplementary Information

Robert W. Schmidt,[†,‡,§] Giulia Giubertoni,[*,‡,§] Federico Caporaletti,[‡,¶] Paul Kolpakov,[‡] Noushine Shahidzadeh,[‡] Freek Ariese,[†] and Sander Woutersen[*,‡]

†*Vrije Universiteit Amsterdam, De Boelelaan 1105, 1081HV Amsterdam, The Netherlands*

‡*University of Amsterdam, Science Park 904, 1098XH Amsterdam, The Netherlands*

¶*Université Libre de Bruxelles, Av. Franklin Roosevelt 50, 1050 Bruxelles, Belgium*

§*These authors contributed equally to the work*

E-mail: g.giubertoni@uva.nl; s.woutersen@uva.nl

Conventional Raman spectra of the component solutions

Figure S1 shows the full Raman spectrum for all three compounds, measured individually in aqueous solution. Raman peaks below 300 cm^{-1} are mainly due to the microscope glass on which the solution samples were placed, due to the use of an inverted Raman microscope.

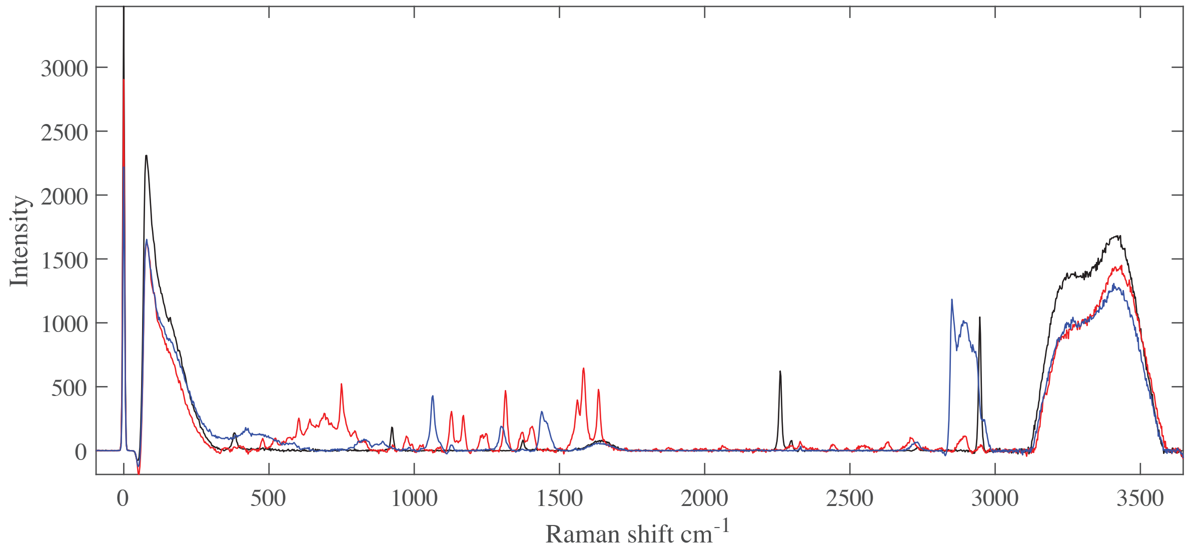

Figure S1: Raman spectrum of acetonitrile (black), cytochrome c (red) and SDS (blue) in water. The spectra were baseline corrected and the cosmic-ray contribution was removed.

.

Singular-Value Decomposition of the raw data

Figure S2 and S3 show the SVDs of the two- and three-component mixed solutions. In each case, the number of significant vectors (i.e. vectors that do not contain pure noise contributions) is equal to the number of components in the mixture.

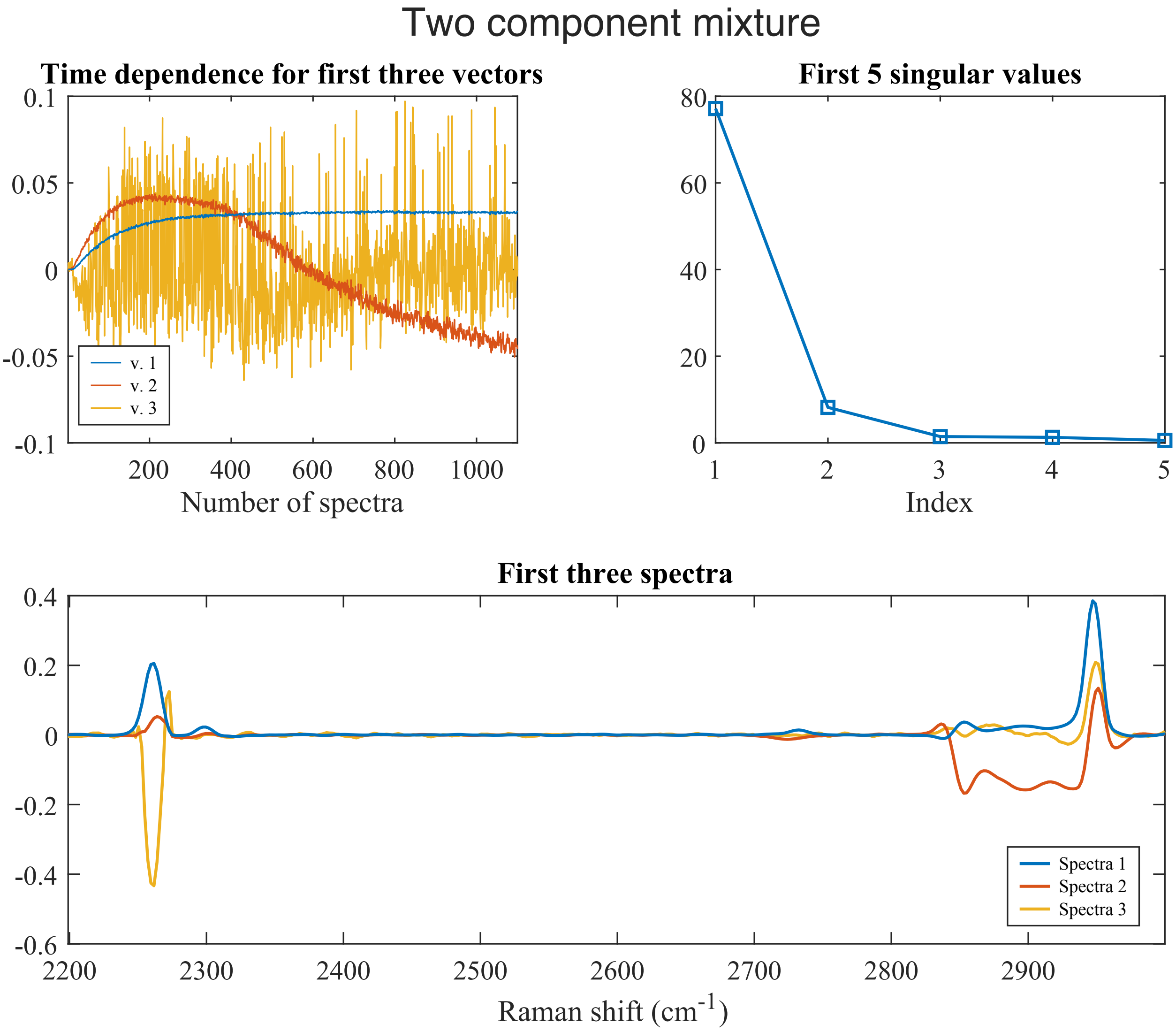

Figure S2: SVD of the raw time-dependent Raman data of the two-component mixed solution.

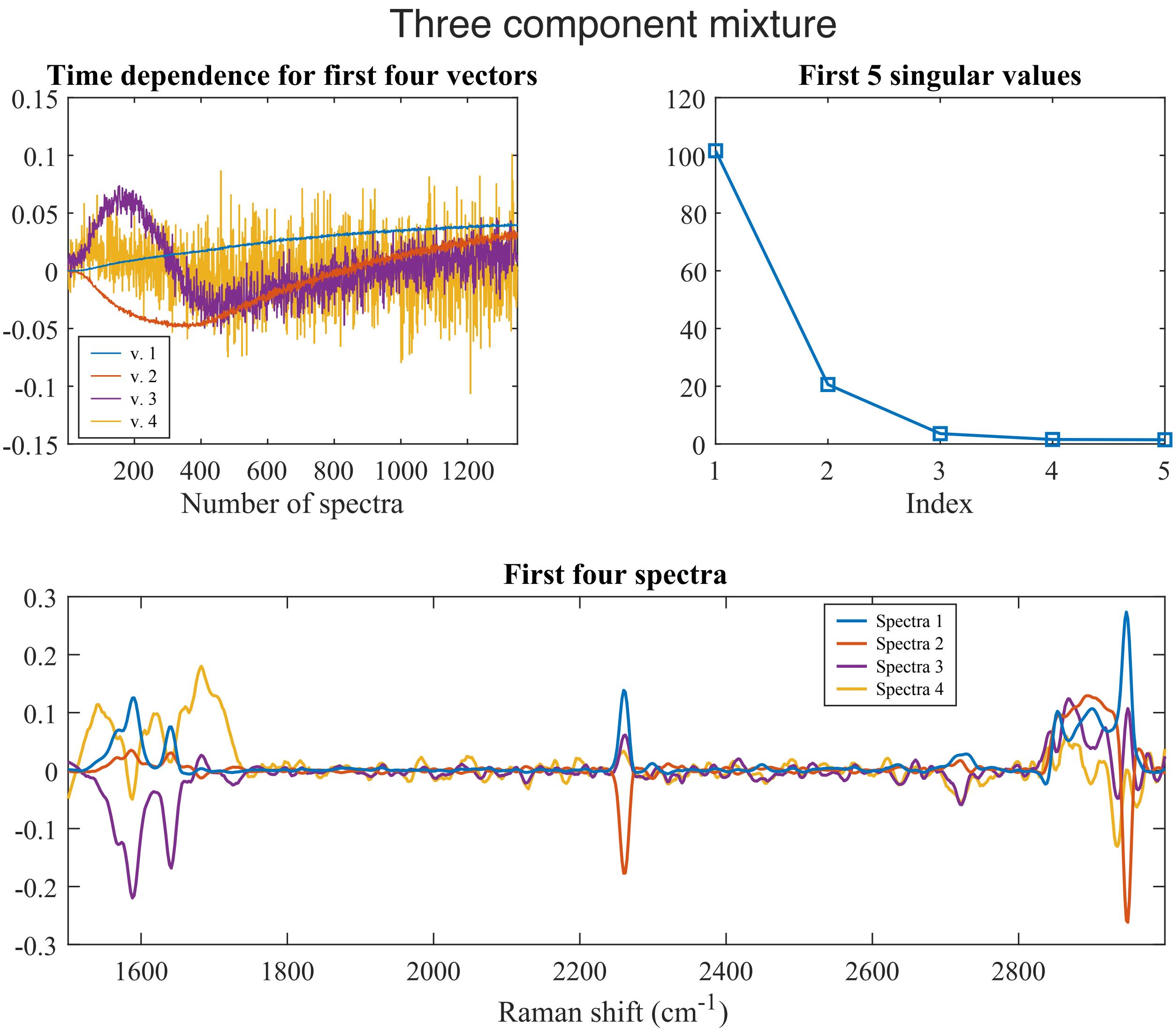

Figure S3: SVD of the raw time-dependent Raman data of the three-component mixed solution.
